# Correction: Limits of Feedback Control in Bacterial Chemotaxis

**DOI:** 10.1371/journal.pcbi.1004069

**Published:** 2014-12-11

**Authors:** The PLOS Computational Biology Staff

There are multiple errors in this article.

In the Results section, subsection Analytical Model of the Drift Velocity as a Function of CheY-P Concentration, there is an error in Equation 3. The term e-t/τ_R0_ is incorrect. It should read 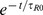 Please view the complete correct equation here: 
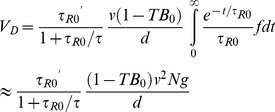
(3)


In the Methods section, subsection Linear Expansion, the inline equation in the first paragraph is incorrect. Please view the complete correct equation here: 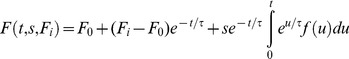



In the Methods section, subsection Linear Expansion, the inline equation on line 30 is incorrect. Please view the complete correct equation here: 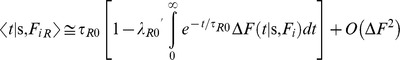



In the Methods Section, subsection Motor Adaptations:

The subscript in the definition of the parameter *k_on_*  = 0.025 s^-1^ is incorrect. The correct subscript should be: *k_off_*  =  0.025 s^-1^

The expression: “Δ*n*  =  4.16 , *ϵ*3,1  =  1.96 to reproduce [19] (Figure 5B insert). *k_off_*  =  0.0063 s^-1^.” is incorrect. The correction expression should be: “Δ*n*  =  2.74, *ϵ*_3,1_  =  2.31 to reproduce [19] (Figure 5A). *k_on_*  =  0.0063s^-1^.”

In the Supporting Information Legends:

In the legend for Figure S4, *k_off_* should be *k_on._*

In the legend for Figure S5, *k_off_* =  0.0013 s^-1^ should be *k_on_* =  0.0013 s^-1^.
