# Supplementary figures and images for: Limits of Feedback Control in Bacterial Chemotaxis
Source: PLoS Comput Biol. 2014 Jun 26;10(6):e1003694. doi: 10.1371/journal.pcbi.1003694 (PMC4072517; doi:10.1371/journal.pcbi.1003694)

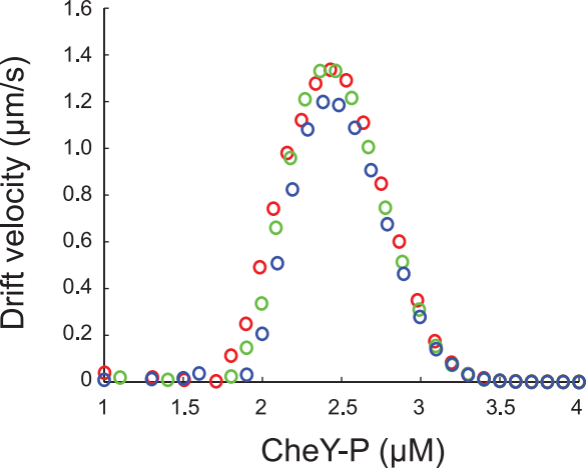

Supplement: Figure S1 — Effect of asymmetric methylation/demethylation rates on drift velocity VD in exponential gradient. Simulated drift velocity VD (average velocity of 10,000 cells between t = 60 and 300 s) as a function of operational CheY-P concentration Ym in a shallow gradient (L0 = 200 µM and g −1 = 5,000 µm) for cells with methylation rates VR = 0.1 s−1 (red), 0.2 s−1 (green), and 0.4 s−1 (blue). (PDF) [file pcbi.1003694.s001.pdf]

**A**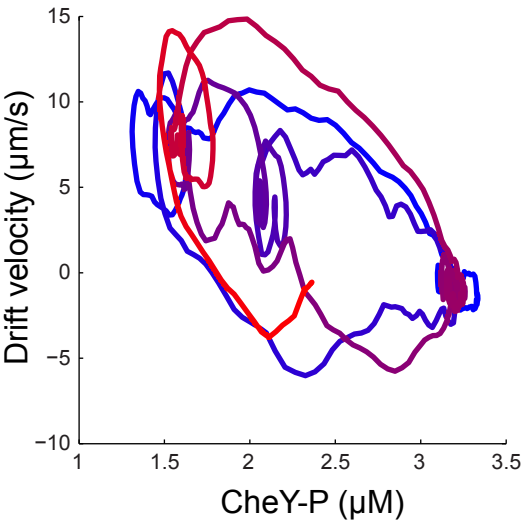**B**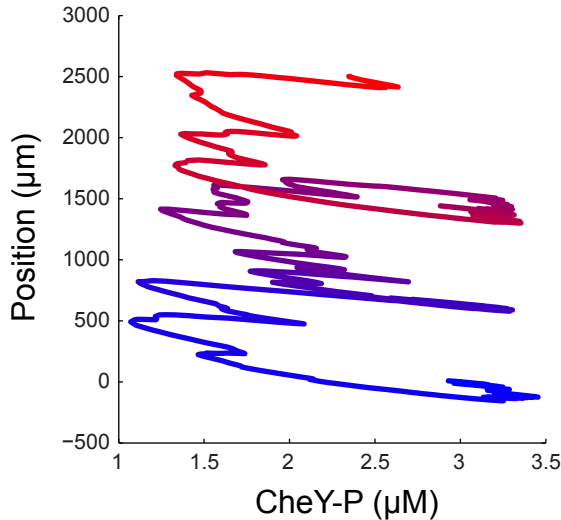

Supplement: Figure S2 — A simulated cell can transition in and out of the non-chemotactic state to reach the high drift velocity state when swimming in a steep gradient of methyl-aspartate (g−1 = 1,000 µm) illustrating the bi-stable behavior of this cell phenotype (Y0 = 3.0 µM and τ = 30 s). A. Single cell drift velocity as a function of its operational CheY-P concentration. When the cell escapes the “trapped” chemotactic state, characterized by a high CheY-P concentration, the behavioral feedback maintains an optimal CheY-P concentration and a high drift velocity. B. Cell position along the gradient as a function of its operational CheY-P concentration. The cell can escape the low drift velocity state and maintain a low CheY-P concentration when running up the gradient. On the other hand, the cell can return to the “trapped” state after a long run down the gradient. The CheY-P concentration and drift velocity were calculated over a moving average window of 10 seconds. The time progression along the trajectory is indicated by the color of the stroke from blue to red. (PDF) [file pcbi.1003694.s002.pdf]

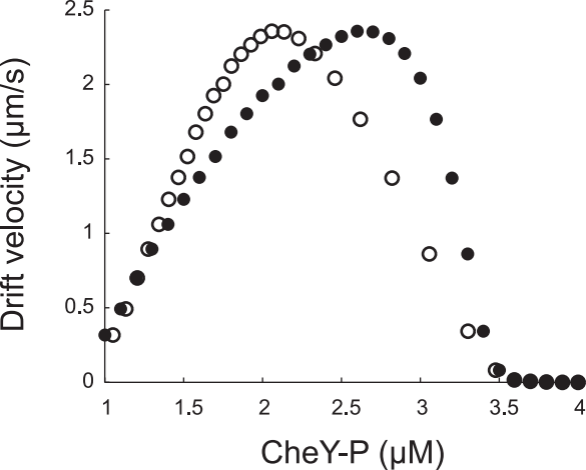

Supplement: Figure S3 — Effect of asymmetric methylation/demethylation rates on drift velocity VD in steep exponential gradient. VD from stochastic simulations (methylation rate VR = 0.1 s−1) as a function of Y0 (filled circles) and Ym (open circles) in exponential gradient of methyl-aspartate (g −1 = 1,000 µm). (PDF) [file pcbi.1003694.s003.pdf]

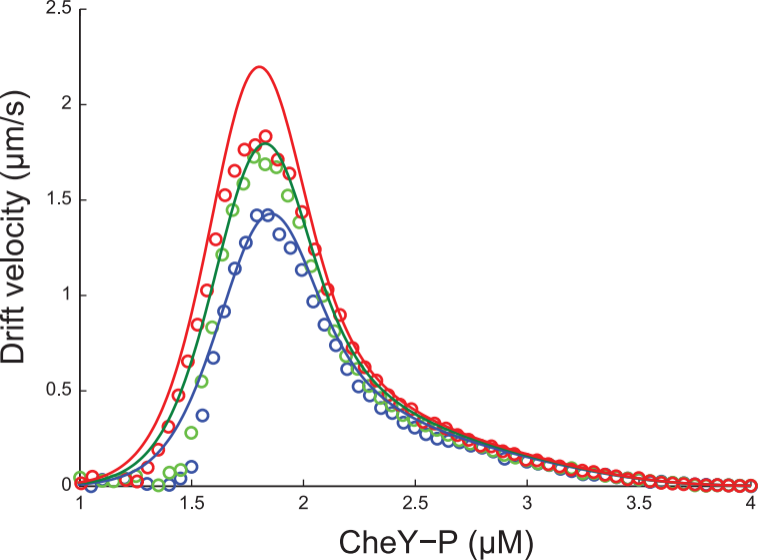

Supplement: Figure S4 — Drift velocity as a function of operational CheY-P when the rate of binding between FliM and the motor is koff = 0.025 s−1. Circles are from simulations. Lines are from analytical solution. Everything is the same as in Figure 5A. The only difference is the value of koff. (PDF) [file pcbi.1003694.s004.pdf]

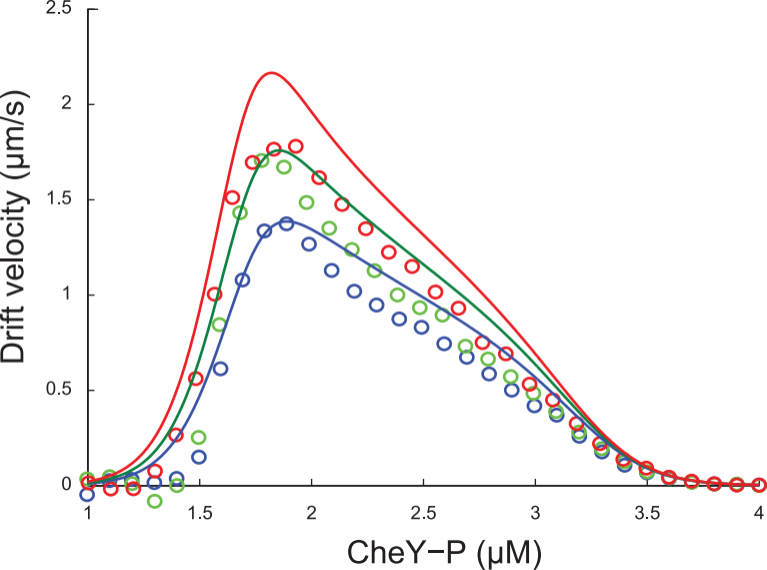

Supplement: Figure S5 — Drift velocity as a function of operational CheY-P when the rate of binding between FliM and the motor is koff = 0.0013 s−1. In this case, the motor does not adapt fast enough to reach quasi-steady state. The analytical solution (lines) makes the approximation that the system is at steady state. (PDF) [file pcbi.1003694.s005.pdf]

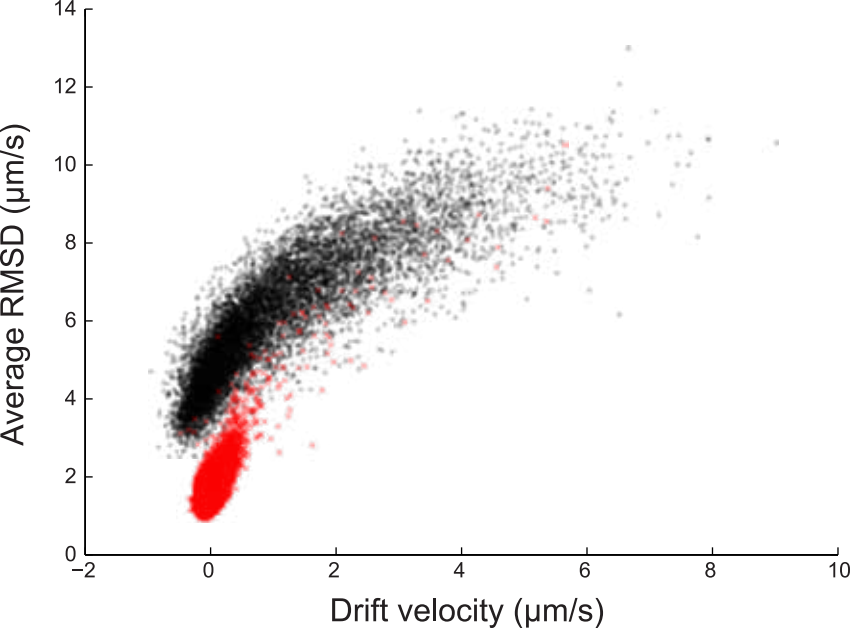

Supplement: Figure S6 — Scatter plot of individual drift velocities (in the direction of the gradient) and root mean square displacements (perpendicular to the gradient) of 10,000 simulated cells with motor adaptation (black) and without motor adaptation (red), with adaptation time τ = 30 s adapted CheY-P concentration Y0 = 3.5 µM, gradient length scale g −1 = 1,000 µm. (PDF) [file pcbi.1003694.s006.pdf]
