# Supplementary material for: Limits of Feedback Control in Bacterial Chemotaxis
Source: PLoS Comput Biol. 2014 Jun 26;10(6):e1003694. doi: 10.1371/journal.pcbi.1003694 (PMC4072517; doi:10.1371/journal.pcbi.1003694)
Supplement: Text S1 — Detailed derivation of the analytical model. (PDF) [file pcbi.1003694.s007.pdf]

Supplementary Information

Mapping of the 2D and 3D problem onto 1 dimension: scaling of drift velocity and rotational diffusion

Assume the cell swims along the direction $\vec{r}$, where $\vec{r}$ is a unit vector in d = 2 or 3 dimensions. Define $s(\theta) = \cos\theta$, where θ is the angle between the direction of motion and the direction of the gradient. Due to rotational diffusion [1], the directional of motion slowly drift away from the original direction during a run. The correlation function is $\langle cos\theta\rangle(t) = \langle \vec{r}(t)\cdot\vec{r}_i\rangle = e^{-(d-1)D_r t}$, where $\vec{r}_i$ is the direction of motion at the beginning of the run. The survival probability distribution of a run along the direction $\vec{r}$ becomes,

$$\mathcal{F}_R\left(t|\theta\right) = e^{-(d-1)D_r t}\cdot e^{-\int_0^t\left(\lambda_R\left(F\left(u,s_i,F_i\right)\right)\right)du} \qquad \text{(S1)}$$

where $F\left(t,s_i,F_i\right) = F_0 + (F_i - F_0)e^{-t/\tau} + e^{-t/\tau}\int_0^t e^{u/\tau}s_i f\left(u\right)du$ and $s_i = \cos\theta_i$ is the cosine of the angle between the direction of the signal gradient and the direction of the cell motion at the beginning of the run. In calculating the change in the free energy we neglected the higher order deviations in the angle of motion. The main effect of rotational diffusion is encapsulated in the first factor, $e^{-(d-1)D_r t}$.

At steady state, we linearize Eq. (S1) around the mean free energy F_m, which is on average lower than the resting free energy F_0.

$$\mathcal{F}_R\left(t|s_i,F_i\right) = e^{-\left((d-1)D_r+\lambda_{Rm}\right)t}\left[1-\lambda_{Rm}^{'}\int_0^t \Delta F\left(\mathrm{u},s_i,F_i\right)du\right] + O\left(\Delta F^2\right) \qquad \text{(S2)}$$

$$\mathcal{F}_T\left(t|s_i,F_i\right) = e^{-\lambda_{Tm}t}\frac{\lambda_{Rm}}{\left(d-1\right)D_r+\lambda_{Rm}} + O\left(\Delta F\right) \qquad \text{(S3)}$$

where $\Delta F = F - F_m$. Integrating over time between 0 and infinity we get the expect run and tumble duration along the direction s_i

$$\left\langle t|s_i,F_i\right\rangle_R \cong \frac{1}{(d-1)D_r+\lambda_{Rm}}\left[1-\lambda_{Rm}^{'}\int_0^\infty e^{-\left((d-1)D_r+\lambda_{Rm}\right)t}\Delta F\left(t,s_i,F_i\right)dt\right] + O\left(\Delta F^2\right) \qquad \text{(S4)}$$

$$\left\langle t|s_i,F_i\right\rangle_T \cong \frac{\lambda_{Rm}/\lambda_{Tm}}{(d-1)D_r+\lambda_{Rm}} + O\left(\Delta F\right) \qquad \text{(S5)}$$

Inserting Eqs. (S4) and (S5) in Eq. (1) the drift velocity is

$$V_D = v\frac{\int_{-1}^{1}\langle t|s_i,F\rangle_{iR}\ s_i\,P(s_i)\,ds_i}{\int_{-1}^{1}\left(\langle t|s_i,F_i\rangle_R+\langle t|s_i,F_i\rangle_T\right)P(s_i)\,ds_i} \tag{S6}$$

where $P(s_i)=2\pi$ in 3D and $(1-s_i^2)^{-1}$ in 2D. We are interested in the first order solution. Therefore only the zeroth order is needed for the denominator: $\frac{1+\lambda_{Rm}/\lambda_{Tm}}{(d-1)D_r+\lambda_{Rm}}\int_{-1}^{1}P(s_i)\,ds_i$.

For the numerator we have to first order in ΔF and noticing that only the terms that are function of s_i are not zero:

$$\frac{-\lambda'_{Rm}}{(d-1)D_r+\lambda_{Rm}}\int_{-1}^{1}ds_i\,s_i^2\,P(s_i)\int_0^\infty dt\,e^{-\left((d-1)D_r+\lambda_{Rm}+\tau^{-1}\right)t}\int_0^t du\,e^{\frac{u}{\tau}}f(u)$$
$$=\frac{\tau'_{Rm}}{\tau_{Rm}}\int_{-1}^{1}ds_i\,s_i^2\,P(s_i)\left[-\frac{e^{-\left(\tau_{Rm}^{-1}+\tau^{-1}\right)t}}{\tau_{Rm}^{-1}+\tau^{-1}}\int_0^t du\,e^{\frac{u}{\tau}}f(u)\Bigg|_0^\infty+\int_0^\infty dt\frac{e^{-\tau_{Rm}^{-1}t}}{\tau_{Rm}^{-1}+\tau^{-1}}f(t)\right]$$

The variation in $f(t)$ depends on the direction of motion

$$f(t)=v\,N\,\partial_x\ \ln[(1+L/K_i)/(1+L/K_a)]$$

The above relation shows that as long as $\int_0^t du\,e^{\frac{u}{\tau}}f(u)$ increases slower than $e^{\left(\tau_{Rm}^{-1}+\tau^{-1}\right)t}$ (the ligand gradient is not steeper than $e^{e^{\tau_{Rm}^{-1}x/v}}$), the numerator becomes

$$\frac{\tau'_{Rm}}{\tau_{Rm}}\int_{-1}^{1}ds_i\,s_i^2\,P(s_i)\int_0^\infty dt\frac{e^{-\tau_{Rm}^{-1}t}}{\tau_{Rm}^{-1}+\tau^{-1}}f(t)$$

Thus, for $K_i << L << K_a$ and exponential gradients $f\approx v\,N\,g$ constant, the drift velocity in 2D/3D is

$$V_D\cong\frac{\tau'_{Rm}}{1+\tau_{Rm}/\tau}\frac{(1-CW_0)v}{d}\int_0^\infty\frac{e^{-t/\tau_{Rm}}}{\tau_{Rm}}f(t)\,dt\approx\frac{\tau'_{Rm}}{1+\tau_{Rm}/\tau}\frac{(1-CW_0)v^2Ng}{d} \tag{S7}$$

which is the same as Eq. (3) in the main text (after the subscript 0 has been replaced by m).

Nonlinear solution

All analytical curves in the paper (lines in Figures 2-5) use the linear approximation around F_0 and F_m as described in the main text and Materials and Methods. Here we describe how to solve Eqs. (1)-(3) keeping the nonlinearity of the rates $\lambda_R(F)$ and $\lambda_T(F)$. Eq. (S16) can be integrated numerically to calculate the stopping time (red and green circles) of the red and green trajectories in Figure 2B. All other analytical curves in the paper (lines in Figures 2-6) use the linear approximation around F_0 and F_m as described in Materials and Methods.

In the 1D representation, the equation for the free energy difference F can be integrated to get $F(t,s,F_i) = \left(1-e^{-t/\tau}\right)\left(F_0 - sf\tau\right) + F_i e^{-t/\tau}$ where $s = \pm 1$ when the cell runs up or down the gradient and $s = 0$ during tumbles. F_i is the initial value. $f \approx v\,N\,g$ is the constant "force" exerted by the gradient.

Inverting we also get the time duration as a function of the free energy:

$$t(F,s,F_i) = \tau \log\left(\frac{F_i + s\,f\,\tau - F_0}{F + s\,f\,\tau - F_0}\right) \qquad \text{(S8)}$$

At steady state the conditional probability densities of the duration t of runs and tumbles are

$$P_{R\to T}\left(t\middle|\mathrm{s},F_{iR}\right) = \lambda_R\left(F\left(t,\mathrm{s},F_{iR}\right)\right) e^{-\int_0^t \left((d-1)D_r + \lambda_R\left(F\left(u,\mathrm{s},F_{iR}\right)\right)\right)du} \qquad \text{(S9)}$$

$$P_{R\to R}\left(t\middle|\mathrm{s},F_{iR}\right) = (d-1)D_r e^{-\int_0^t \left((d-1)D_r + \lambda_R\left(F\left(u,\mathrm{s},F_{iR}\right)\right)\right)du} \qquad \text{(S10)}$$

$$P_{T\to R}\left(t\middle|F_{iT}\right) = \lambda_T\left(F\left(t,0,F_{iT}\right)\right) e^{-\int_0^t \lambda_T\left(F\left(u,0,F_{iT}\right)\right)du} \qquad \text{(S11)}$$

where $s = \pm 1$ and the first two probability densities correspond to runs that terminate into a tumble and into a run of opposite direction, respectively. F_{iR} and F_{iT} are the values of F at the beginning of a run and a tumble, respectively. Noting that $dt = -\tau \dfrac{dF}{F + s\,f\,\tau - F_0}$ we also get

$$P_{R\to T}\left(F\middle|\mathrm{s},F_{iR}\right) = P_{R\to T}\left(t\left(F,s,F_{iR}\right)\middle|\mathrm{s},F_{iR}\right)\frac{-\tau}{F + sf\tau - F_0} = \frac{-\tau\,\lambda_R\left(F\right)}{F + sf\tau - F_0} e^{\tau\int_{F_{iR}}^{F}\frac{(d-1)D_r + \lambda_R(F')}{F' + s\,f\,\tau - F_0}dF'}$$

(S12)

$$P_{R\to R}\left(F\middle|s,F_{iR}\right)=P_{R\to R}\left(t\left(F,s,F_{iR}\right)\middle|s,F_{iR}\right)\frac{-\tau}{F+sf\tau-F_0}=\frac{-\tau\,(d-1)D_r}{F+sf\tau-F_0}e^{\tau\int_{F_{iR}}^{F}\frac{(d-1)D_r+\lambda_R(F')}{F'+s\,f\,\tau-F_0}dF'}$$

(S13)

$$P_{T\to R}\left(F\middle|F_{iT}\right)=P_{T\to R}\left(t\left(F,0,F_{iT}\right)\middle|F_{iT}\right)\frac{-\tau}{F-F_0}=\frac{-\tau\,\lambda_T\left(F\right)}{F-F_0}e^{\tau\int_{F_{iT}}^{F}\frac{\lambda_T\left(F'\right)}{F'-F_0}dF'} \quad \text{(S14)}$$

The probability density to have free energy F_e at the end of a run and tumble cycle is then

$$P\left(F_e\middle|F_{iR}\right)=\frac{1}{2}\sum_{s=\pm1}\left(P_{R\to R}\left(F_e\middle|s,F_{iR}\right)+\int_{-\infty}^{\infty}P_{T\to R}\left(F_e\middle|F_{iT}\right)P_{R\to T}\left(F_{iT}\middle|s,F_{iR}\right)dF_{iT}\right)$$

(S15)

At steady state we must have $P\left(F_e\right)=\int_{-\infty}^{\infty}P\left(F_e\middle|F_{iR}\right)P\left(F_{iR}\right)dF_{iR}$ equal to $P\left(F_{iR}\right)$, which given $P\left(F_e\middle|F_{iR}\right)$ defines $P\left(F_{iR}\right)$. The average run and tumble durations are then

$$\left\langle t\middle|s\right\rangle_R=\int_0^{\infty}dt\,t\int_{-\infty}^{\infty}dF_{iR}\left(P_{R\to R}\left(t\middle|s,F_{iR}\right)+P_{R\to T}\left(t\middle|s,F_{iR}\right)\right)P\left(F_{iR}\right) \quad \text{(S16)}$$

$$\left\langle t\middle|s\right\rangle_T=\int_0^{\infty}dt\,t\int_{-\infty}^{\infty}dF_{iR}P\left(F_{iR}\right)\int_{-\infty}^{\infty}dF_{iT}P_{T\to R}\left(t\middle|F_{iT}\right)P_{R\to T}\left(F_{iT}\middle|s,F_{iR}\right) \quad \text{(S17)}$$

We obtain the drift velocity

$$V_D=\frac{\frac{1}{2}\sum_{s=\pm1}s\left\langle t\middle|s\right\rangle_R}{\frac{1}{2}\sum_{s=\pm1}\left(\left\langle t\middle|s\right\rangle_R+\left\langle t\middle|s\right\rangle_T\right)}\frac{v}{d} \quad \text{(S18)}$$

Effect of asymmetric methylation/demethylation rates

Experimental data shows the methylation/demethylation rates for receptor adaptation are asymmetric [2]. The rate of change of methylation catalyzed by CheR and CheB is usually described as

$$\frac{dm}{dt}=V_R\frac{1-a}{1-a+K_R}-V_B(a)\frac{a}{a+K_B} \quad \text{(S19)}$$

where V_R and $V_B(a)$ are the rates of methylation and demethylation; and K_R and K_B are the constants for each reactions. Experimentally, people found that the asymmetry of

methylation/demethylation rates is not significant until $a > a_B$, where $a_B \approx 0.78$ measured in [2]. $V_B(a)$ is a piece-wise linear function: $V_B(a) = V_{B,0}(1 + k_B \Theta(a - a_B) \frac{a - a_B}{1 - a})$, where $\Theta(x)$ is a unit step function ($\Theta(x) = 1$ only if $x>0$).

For most of the dynamic range of CheY-P level we are interested in $\Theta(a - a_B)$ will be zero and V_B will be approximately constant. Thus variations in the rate of demethylation should not affect much drift velocity and optimal CheY-P level. To verify this, we implemented Eq. (S19) into our stochastic simulations of individual cells, with K_R=0.43, K_B=0.3 and k_B=2.7 [2]. To vary the adapted CheY-P level we varied V_R and $V_{B,0}$ since their ratio determines the adapted CheY-P level. Given these definitions the effective adaptation time scale τ_{eff} obtained by linearizing equation (S19) reads $\tau_{eff} = \frac{a_0(a_0 + K_B)(1 - a_0 + K_R)^2}{\left[a_0^2 K_R + ((1 - a_0)^2 + K_R)K_B\right]V_R}$, where a_0 is the adapted activity of the receptor (corresponding to adapted CheY-P level Y_0 in this case) . As shown in Fig. S1, the optimal CheY-P level in shallow gradient remains at the same position with respect to the motor response curve as in Fig. 2A. While the cells drifts in the steep gradient with slow methylation and demethylation rates, the behavior feedback will still push the system to a bifurcation (Fig. S2).

Adapted motor response curve

The motor adaptation is considered in this study by assuming that the number of FliM molecules in the motor changes as a function of the CW bias of the motor. At steady state, where dn/dt = 0, the relation between CW bias and number of FliM, n, is given by

$$CW(n) = \frac{(n - n_1 + \Delta n)(n_2 - n)\frac{k_{on}}{k_{off}}}{\left(1 + \frac{k_{on}}{k_{off}}\right)(n_2 - n)(n - n_1) + \Delta n\left[(n_2 - n)\frac{k_{on}}{k_{off}} + (n - n_1)\right]} \quad \text{(S20)}$$

Eq. (S20) together with the CW bias response function, $CW(Y_m, \varepsilon_3) = 1/(1 + e^{2G(Y_m, \varepsilon_3)})$ and the linear relation between free energy ε_3 and the number of FliM n: $\varepsilon_3 = \varepsilon_{3,1}(n - n_0) + \varepsilon_{3,0}$, gives ε_3 as a function of Y_m. The adapted motor response curve $CW(Y_m, \varepsilon_3)$ is calculated then to fit the experimental data [3] with parameters Δn and $\varepsilon_{3,1}$.

Note that for Figures 4 and 5 of the main text, k_{on} was chosen as 0.0063 s^{-1}, so that the CW bias that the motor adapts to is 0.2, which is the average CW bias measured experimentally in wild type population of *E. coli* selected for swimming on agar plates [4]. We also examined what would happen if we changed the CW bias that the motor adapts to. For $k_{on} = k_{off}$ =0.025 s^{-1} the effect of motor adaptation on the drift velocity curve is most visible for Y_m between 2.5 and 3.5 µM (Fig. S3) whereas it is between 2 and 3 µM when k_{on} = 0.0063 s^{-1} (Fig. 4A). We also simulated the case where the CW bias that the motor adapts to is 0.05 (here k_{on} = 0.0013 s^{-1})

which results in a flat region of the drift velocity curve as a function of Y_m around the optimal operational CheY-P level (~2 μM) (Fig. S4). In this case, because k_{on} is so small the adaptation time of the motor is very long and the motor does not reach steady state during the simulation. This explains the slight discrepancy with the analytical solution, which assumes steady state of the motor.

References

1. Locsei JT (2007) Persistence of direction increases the drift velocity of run and tumble chemotaxis. Journal of Mathematical Biology.
2. Shimizu TS, Tu Y, Berg HC (2010) A modular gradient-sensing network for chemotaxis in Escherichia coli revealed by responses to time-varying stimuli. Mol Syst Biol 6: 382.
3. Cluzel P, Surette M, Leibler S (2000) An ultrasensitive bacterial motor revealed by monitoring signaling proteins in single cells. Science (New York), NY 287: 1652-1655.
4. Park H, Pontius W, Guet CC, Marko JF, Emonet T, et al. (2010) Interdependence of behavioural variability and response to small stimuli in bacteria. Nature 468: 819-823.
